# Tick Ecdysteroid Hormone, Global Microbiota/*Rickettsia* Signaling in the Ovary versus Carcass during Vitellogenesis in Part-Fed (Virgin) American Dog Ticks, *Dermacentor variabilis*

**DOI:** 10.3390/microorganisms9061242

**Published:** 2021-06-08

**Authors:** Loganathan Ponnusamy, Haley Sutton, Robert D. Mitchell, Daniel E. Sonenshine, Charles S. Apperson, Richard Michael Roe

**Affiliations:** 1 Department of Entomology and Plath Pathology, North Carolina State University, Raleigh, NC 27695, USA; lponnus@ncsu.edu (L.P.); Haley.Sutton@ncagr.gov (H.S.); Mitchell.Robert@epa.gov (R.D.M.III); charles_apperson@ncsu.edu (C.S.A.); 2 Comparative Medicine Institute, North Carolina State University, Raleigh, NC 27695, USA; 3 North Carolina Department of Agriculture and Consumer Services, Raleigh, NC 27601, USA; 4 Office of Pesticide Programs, Invertebrate and Vertebrate Branch 1, Registration Division, U.S. Environmental Protection Agency, 1200 Pennsylvania Ave., Washington, DC 20460, USA; 5 Department of Biological Sciences, Old Dominion University, Norfolk, VA 23529, USA; daniel.sonenshine@nih.gov

**Keywords:** *Dermacentor variabilis*, ecdysteroid, *Rickettsia* sp., *Francisella* sp., qPCR, microbiota

## Abstract

The transovarial transmission of tick-borne bacterial pathogens is an important mechanism for their maintenance in natural populations and transmission, causing disease in humans and animals. The mechanism for this transmission and the possible role of tick hormones facilitating this process have never been studied. Injections of physiological levels of the tick hormone, 20-hydroxyecdysone (20E), into part-fed (virgin) adult females of the American dog tick, *Dermacentor variabilis*, attached to the host caused a reduction in density of *Rickettsia montanensis* in the carcass and an increase in the ovaries compared to buffer-injected controls. This injection initiates yolk protein synthesis and uptake by the eggs but has no effect on blood feeding. *Francisella* sp. and *R. montanensis* were the predominant bacteria based on the proportionality in the carcass and ovary. The total bacteria load increased in the carcass and ovaries, and bacteria in the genus *Pseudomonas* increased in the carcass after the 20E injection. The mechanism of how the *Rickettsia* species respond to changes in tick hormonal regulation needs further investigation. Multiple possible mechanisms for the proliferation of *R. montanensis* in the ovaries are proposed.

## 1. Introduction

Ticks are obligatory hematophagous arthropods known for their ability to act as reservoirs and vectors for infectious microbial agents that cause human illnesses, including Lyme disease, Rocky Mountain spotted fever (RMSF), spotted fever group (SFG) rickettsiosis, human granulocytic anaplasmosis, Babesiosis, and human monocytic ehrlichiosis; these diseases cause significant morbidity in the United States [1,2]. The most important pathogens are the spirochete, *Borrelia burgdorferi* sensu stricto, and the bacterium, *Rickettsia rickettsii*, responsible for Lyme disease and RMSF, respectively [3,4]. In the continental US, RMSF is the most severe of the SFG rickettsioses and is often fatal [5,6,7]. The principal arthropod vectors are ixodid ticks, namely, the Rocky Mountain wood tick, *Dermacentor andersoni*, in the western US, the American dog tick, *D. variabilis,* in the eastern half and west coast of the US, and the brown dog tick, *Rhipicephalus sanguineus*, in focal areas of Arizona, US [8].

Pathogens like rickettsiae are passed from one generation to the next by the female to her eggs [9]. This vertical, transovarial transmission is critical in maintaining infected populations of the American dog tick and the spread of pathogenic SFG *Rickettsia* and RMSF to people [10]. Our knowledge of the mechanism for transovarial infection involving microorganisms in insects is minimal and is even less in ticks. In insects, the vertical transmission of the rice stripe virus (RSV) in the small brown planthopper, *Laodelphax striatellus*, occurs when RSV hitchhikes by binding to the female yolk protein and enters developing oocytes through endocytosis [11]. Presumably, there must be viral surface proteins that promote yolk protein binding.

Vitellogenins (Vg) in insects and ticks are hemolymph precursors of the yolk protein in the egg, vitellin (Vn), a phosphoglycolipoprotein utilized in oviparous animals to provide nutrition for the developing embryo. Multiple vitellogenin genes are found in many animals, including frogs [12], chickens, nematodes [13], and arthropods, e.g., the fruit fly, *Drosophila melanogaster* [14], yellow fever mosquito, *Aedes aegypti* [15] and ticks [16,17,18]. In replete (i.e., fully engorged), mated adult female *D. variabilis*, Vg is synthesized in the fat body and midgut, is secreted into the hemolymph, captured from the hemolymph by Vg receptors (VgRs) on the oocytes and endocytosed into developing oocytes across clathrin-coated pits on the oocyte’s outer surface [16,18,19,20,21]. The VgR message has been identified in a variety of tick species, including *D. variabilis* [17], *Haemaphysalis longicornis* [22], *Amblyomma hebraeum* [23], *Rhipicephalus microplus* and *R. appendiculatus* [24]. The amino acid sequence of the *D. variabilis* Vn and VgR has a high similarity to the same from *R. microplus*, *Ixodes scapularis*, and *A. hebraeum* and even to other animals outside of the Acari. We found that silencing VgR in the American dog tick by RNAi (RNA interference) prevented Vn deposition [17] as was also the case for *R. microplus* [24].

In ticks, rickettsiae pass from infected to uninfected cells through actin-based bridges or mechano-transduction [25,26]; however, the mechanism of movement to the tick egg has never been studied. Silencing VgR in the tick, *H. longicornis*, blocked the transmission of the protozoan, *Babesia gibsoni*, from the midgut into oocytes [22] and egg development. *B. gibsoni* causes canine babesiosis, a disease in dogs where the symptoms range from mild fever and lethargy to multi-organ failure and death [27]. Hussein et al. [28] also found that silencing VgR in *R. microplus* blocked the transmission of *B. bovis* while at the same time prevented egg development. No research is available on the tick bacteria microbiome in the ovary during female reproduction.

The hormonal regulation of vitellogenesis in ticks is controlled by neuropeptides from the synganglion (the brain and central nerve chord) that initiate the synthesis and release into the hemolymph of ecdysteroid hormones by the epidermis [16,18,21,29]. These ecdysteroids then initiate the synthesis and secretion of Vg from the fat body and midgut into the hemolymph [30,31]. There are a number of other synganglion neuropeptide hormones described in ticks during reproduction including those in insects that regulate molting. Adult ticks do not molt. The mevalonate pathway is also found in the synganglion, leading to part of the juvenile hormone (JH) synthesis pathway. No JH has been found in ticks like that in insects. In insects, the JH regulates egg development, but this is not the case in ticks. We found that the injection of 20-hydroxyecdysone at physiological levels into part-fed female, virgin *D. variabilis* adults still attached to a rabbit, initiated vitellogenesis, the synthesis of VgR, and the development of vitellogenic eggs and normal ovary growth [29]. These injections did not initiate feeding to repletion or drop off from the host. Egg development followed by detachment from the host and oviposition is normally initiated when the part-fed female mates and then feeds to repletion.

The role of the endocrine system in the regulation of animal microbiomes in general has received minimal attention, and there is no research in ticks. The role of the endocrine system in transovarial transmission of bacteria in ticks has also not been studied. The aim of this study is to determine whether ecdysteroids that regulate oocyte development also regulate the overall bacterial microbiota, especially *Rickettsia* sp., *Francisella* sp. and possibly other bacteria in the body versus the ovary of part-fed, virgin female adult *D. variabilis* ticks, in the absence of mating and the influence of blood feeding.

## 2. Materials and Methods

### 2.1. Ticks and In Vivo Injection of Ecdysteroids

Unfed (virgin) female adults of the American dog tick were purchased from the Department of Entomology at Oklahoma State University, Stillwater, OK, USA, maintained and blood fed as described previously [32], and used directly for experimentation. Adult ticks were confined within plastic capsules attached to New Zealand white rabbits, Oryctolagus cuniculus, for feeding to the part-fed stage. The steroid hormone, 20-hydroxyecdysone (hereafter referred to as 20E; Sigma Chemical Co., St. Louis, MO, USA) was diluted into 0.15 M pH 7.0 phosphate-buffered saline (0.13 M NaCl) (=control buffer) and injected into the bodies of partially fed (virgin) female adults following the method described by Thompson et al. [29]. Technical 20E and 20E diluted into buffer were stored at −20 °C in the dark until needed. Each female received a dose of 1000 ng of 20E on day 4 after attachment to the host. Controls were injected with control buffer only. All injections were performed while female *D. variabilis* ticks were attached to the host; the attached ticks were collected 2 d post injection from the host for microbiota and *Rickettsia* analysis. All use of animals in this study was carried out under protocols approved by the ODU Institutional Animal Care and Use Committee (Animal Welfare Assurance Number: A3172-01), specifically protocol #10-032 for rabbits.

### 2.2. Tissue Dissection and DNA Extraction

Ticks were washed once in 0.5% bleach and 5 times with sterile phosphate-buffered saline (PBS) (10 mM NaH_2_PO_4_, 1.8 mM KH_2_PO_4_, 140 mM NaCl, and 2.7 mM KCl, pH 7.4). The ovary was dissected from each tick body leaving the carcass (the body without the ovary), and total genomic DNA was extracted separately from each carcass and its corresponding ovary for each sample. Extractions were conducted immediately after the dissections. Eight ticks were injected with 20E and eight with control buffer. Total genomic DNA was isolated from the above samples using the phenol-chloroform methods described by Ponnusamy et al. [33]. DNA quality and quantity were assessed using the NanoDrop 1000 Spectrophotometer (Thermo Fisher Scientific, Waltham, MA, USA). Total genomic DNA from the ovary and the corresponding carcass for each sample was normalized to a concentration of 50 ng/µL and stored at −20 °C.

### 2.3. Microbiota Composition

To determine the total species composition of the bacterial communities in the 20E injected females versus control samples, we used a 16S rRNA gene sequencing approach. DNA was pooled from 4 ticks (chosen at random) and duplicated for ovaries versus the carcass for the 20E versus the control buffer injections for Illumina sequencing. The samples were subjected to 16S rRNA gene amplification of the V3–V4 hypervariable region of the bacteria genomes [34] for sequencing according to the Illumina 16S Metagenomic Sequencing Library Preparation Guide (part number 15044223, revision B). Libraries constructed were quantified with Quant-iT PicoGreen (Molecular Probes, Inc., Eugene, OR, USA), normalized, and then pooled prior to sequencing. Library sequencing was performed at the Microbiome Core Facility, School of Medicine, University of North Carolina, Chapel Hill, NC, USA.

### 2.4. Bioinformatics Data Processing

The initial processing for sequencing was completed using Quantitative Insights Into Microbial Ecology (QIIME, version 1.9.0) [34], an open source software pipeline (http://www.qiime.org/ (accessed on 9 April 2019)). Paired-end reads (V3–V4 regions) were merged using fastq-join with the default QIIME parameters. Demultiplexing was executed using default QIIME settings, which eliminate the reads with an average Phred quality score less than 20. De novo chimera detection and deletion were executed with USEARCH 6.1 (http:/www.drive5.com/userach/ (accessed on 4 May 2019)) [35]. Sequences were trimmed, denoised and each unique bacterial sequence (97% sequence identity) designated as an OTU (operational taxonomy unit) [34]. Sequences were matched against the Greengenes version 13.8 database (http://greengenes.secondgenome.com; accessed on 14 August 2019) using UCLUST [36]. Sequences were aligned against the Greengenes reference database using Python Nearest Alignment Space Termination (PyNAST) [37] filtered to remove gaps.

### 2.5. Quantitative PCR (qPCR) for Total Bacteria

We also conducted qPCR to quantify the total bacteria load in our samples following the method of Lazarevic et al. [38]. In brief, the qPCR used the 357F/518R primer pair that targets the V3 region of the 16S rRNA gene and the host housekeeping gene, glyceraldehyde 3-phosphate dehydrogenase (GAPDH) as the reference gene [39]. The amplification was conducted in five technical replicates for each tick sample, each in a 10 µL reaction mixture containing 2 µL (100 ng) of genomic DNA, 5 µL 2X SYBR Green qPCR Master Mix (BioRad, Hercules, CA, USA ) and 0.5 µM primer. Reactions were performed in a CFX384 real-time PCR machine (Bio-Rad, Hercules, CA, USA), with an initial denaturation at 95 °C for 3 min, followed by 35 cycles at 94 °C for 10 s, 55 °C for 30 s and 68 °C for 30 s. To calculate burden, the efficiency-corrected *Cq* values for the total bacteria were divided by that for the host GAPDH [39].

### 2.6. Quantification of Rickettsia by qPCR

To measure relative abundance of *Rickettsia* spp., qPCR was performed using RCK/23-5N1F and RCK/23-5N1R primers for the 23S-5S ITS gene [40] and the GAPDH reference gene [39]. A total of 2 μL of the isolated DNA (100 ng) from each tick sample was added to the qPCR mix as described, followed by thermal cycling and detection. Reactions were performed in a CFX384 real time PCR machine (Bio-Rad), with an initial denaturation at 95 °C for 3 min, followed by 40 cycles at 94 °C for 10 s, 55 °C for 15 s and 72 °C for 15 s. Five technical replicates were used for each sample. Nuclease-free sterile water instead of the template was used as a blank control. The melting curve temperature analysis ranged from 65 °C to 95 °C at 0.5 °C increments to verify each amplified product’s melting temperature. qPCR assays were conducted using the SYBR Green quantitative PCR contact Bio-Rad CFX384 real time PCR system. To calculate burden, the efficiency-corrected *Cq* values for the genus *Rickettsia*-specific 23S-5S were divided by that for the host housekeeping gene (GAPDH). The target gene specificity of qPCR amplicons was confirmed by melting curve analysis and further confirmed using agarose gel-based electrophoresis.

### 2.7. Sequencing and Phylogenetic Analyses of Rickettsia

To verify the species of *Rickettsia* from our 23S-5S IGS amplicon from *D. variabilis*, we sequenced the samples using the RCK/23-5N1F primer [40]. A total of 3 samples were Sanger sequenced at Eton Bioscience, Inc. (Research Triangle Park, NC, USA). The obtained DNA sequences were compared for similarity to the *Rickettsia* sequences deposited in NCBI database using BLAST. Phylogenies were created to assess the 23S-5S gene sequence variability of the bacteria within their clades. The 23S-5S sequences from ticks were aligned with sequences deposited in GenBank using Clustal X, version 2.0 [41], and a phylogenetic analysis was conducted using the neighbor-joining method [42]. The evolutionary distance was calculated using the Kimura’s two-parameter model [43], and bootstrap analysis with 1000 iterations was carried out with the MEGA X software package [44].

### 2.8. Data Analysis

All qPCR assays were performed in five technical replicates for each sample. Precautions were taken to ensure that each technical data set fell within a 0.5 threshold cycle (*Cq*). No-template controls were used to assess run reliability and cross-contamination. There was no detectable amplification product from no-template controls in any of the qPCR assays. The amplification proficiency of the whole qPCR assays was 100%. All data are expressed as mean values ± SEM. The normalized fold difference levels were determined using Bio-Rad software (Bio-Rad CFX MANAGER v.3.1). The fold increase was considered statistically significant if a *p*-value of <0.05 was obtained when compared with the buffer control. For comparisons between the carcass and ovary, fold increase was considered statistically significant if a *p*-value of <0.05 was obtained between ovary and carcass.

## 3. Results

### 3.1. Overall Distribution of Bacteria within Different Organs

**Figure 1:**
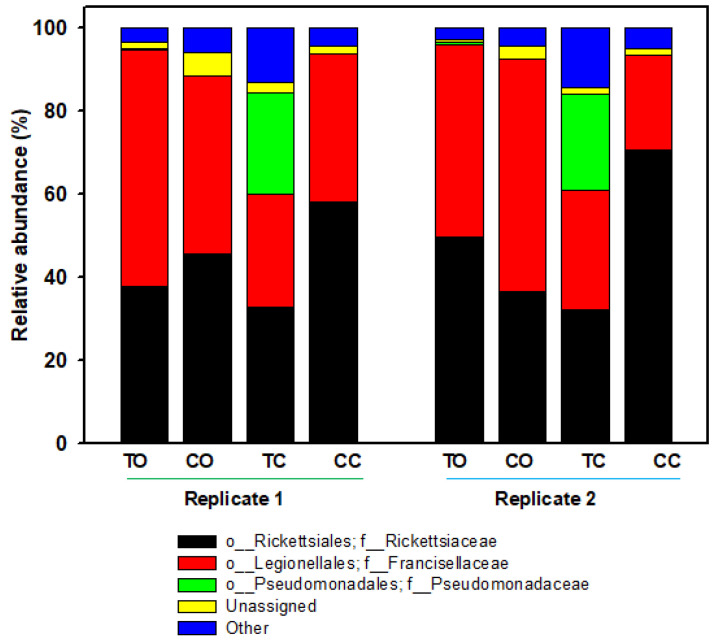
Relative abundance of major bacteria at the family level in part-fed (virgin) *Dermacentor variabilis* ovaries versus carcass for 20E versus control buffer injected ticks. Bars represent the proportions of each taxa. “Other taxa” = all other taxa with relative abundance <1% over the total number of reads. Abbreviations: TO, ovaries from ticks injected with 20-hydroxyecydsone (20E); CO, ovaries from ticks injected with control buffer; TC, carcass from ticks injected with 20E; CC, carcass from ticks injected with control buffer.

To determine the bacterial species composition in different samples, we examined the bacterial 16S rRNA V3 to V4 gene. Among the samples examined, 327,321 sequences were generated after quality filtering. The number of reads varied among samples (minimum of 27,495 and maximum of 63,601). Notably, the bulk of Illumina sequences were from two genera in the family Rickettsiaceae (45.48%) and Francisellaceae (39.50%) comprising 84.98% of all the OTUs (Figure 1, Table 1). The abundance of family Pseudomonadaceae was 6.04%. The remainder of unassigned bacteria and “others” (relative abundance <1%) was 8.5%.

At the treatment level, we found genus *Rickettsia* with relative mean abundances of 43.82% (22,507 reads) in the 20E-treated ovaries and 41.24% (12,803 reads) in the corresponding control. In the carcass, we found the genus *Rickettsia* with relative mean abundances that were 32.51% (10,135 reads) in the 20E-treated ticks and 64.38% (38,957 reads) in the control. A similar trend was also found with the genus *Francisella*. Genus *Pseudomonas* was mostly found in the 20E-treated carcass (23.76%) compared to the control (0.008%; Table 1).

### 3.2. Bacterial Quantification

**Figure 2:**
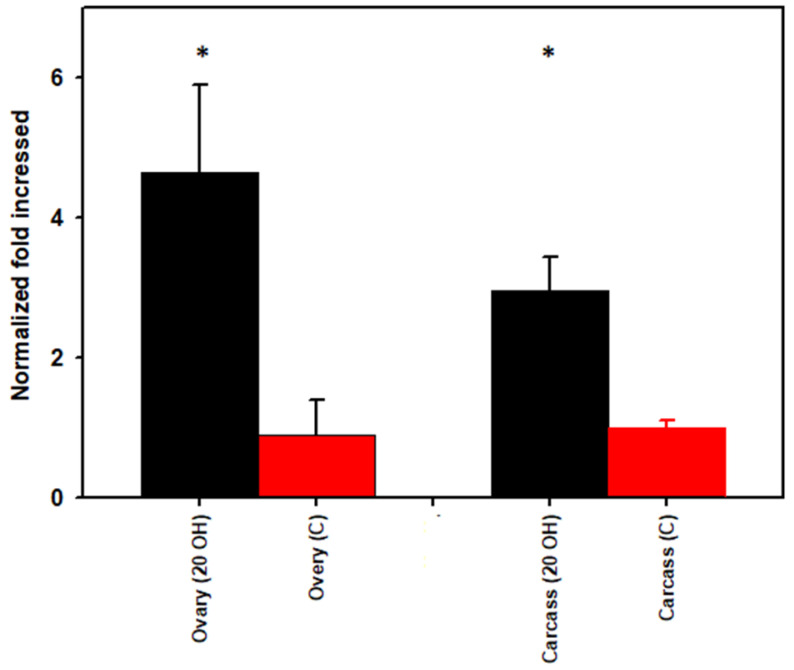
Total bacterial load (qPCR using the 16S gene target) in part-fed (virgin) *Dermacentor variabilis* ovaries versus carcass for 20-hydroxyecdysone (20E) versus control buffer (C) injected ticks. Mean fold change ± SEM, * *p* < 0.05.

The mean threshold for qPCR cycle numbers (*Cq*) for ovaries injected with 20E versus the control buffer were 18.84 and 21.25, respectively. The mean *Cq* for carcass injected with 20E was 17.90 and for the control, 19.04. We observed a significant treatment effect (*p* = 0.0001); total bacteria were higher (4.6-fold) in the ovary and 2.9-fold higher in the carcass in 20E-injected females than in PBS-injected control ticks (Figure 2).

### 3.3. Rickettsia Quantification

**Figure 3:**
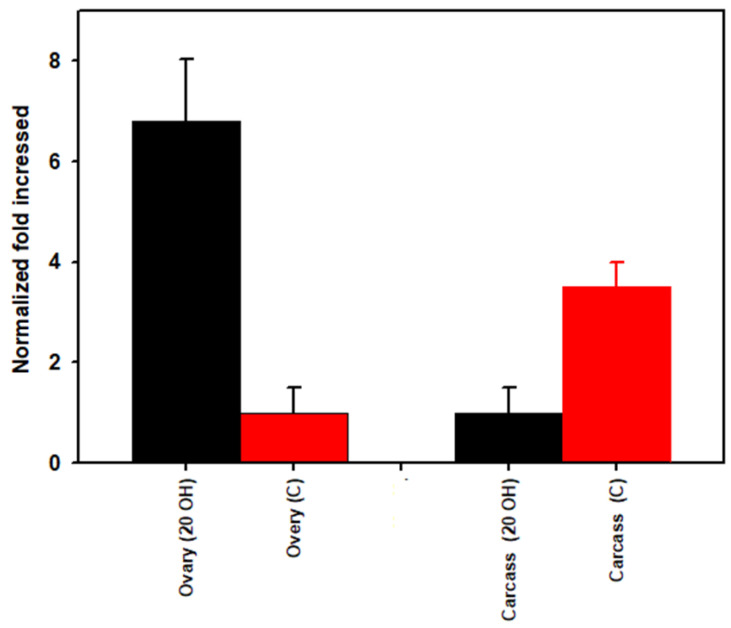
*Rickettsia* spp. load (qPCR using the 23S-5S gene target) in part-fed (virgin) *Dermacentor variabilis* ovaries versus carcass for 20-hydroxyecdysone (20E) versus control buffer (C) injected ticks. Mean fold change ± SEM.

**Figure 4:**
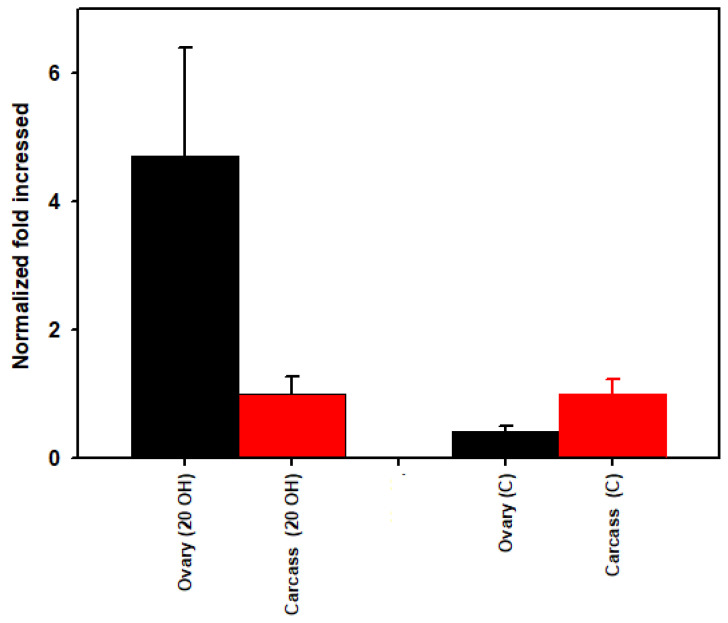
*Rickettsia* spp. load (qPCR using the 23S-5S gene target) in part-fed (virgin) *Dermacentor variabilis* ovaries versus carcass for 20-hydroxyecdysone (20E) versus buffer (C) injected ticks. Mean fold change ± SEM.

The mean threshold quantification cycle numbers (*Cq*) for ovaries injected with 20E versus the control buffer were 20.29 and 22.49, respectively. *Cq* values for carcass for 20E versus the control was 21.87 and 20.56, respectively. The density of *Rickettsia* spp. was higher (6.8-fold) in the ovary and 3.5-fold lower in the carcass after 20E injection compared to the buffer control (Figure 3). When we compare the ovary versus carcass for the 20E injected ticks, *Rickettsia* spp. density was 4.7-fold higher in the ovary than the carcass and the reverse occurred in the buffer injected ticks where the ovary was 0.42-fold lower than the carcass (Figure 4).

### 3.4. Sequencing of 23S-5S Gene and Phylogenic Analyses of Rickettsia

**Figure 5:**
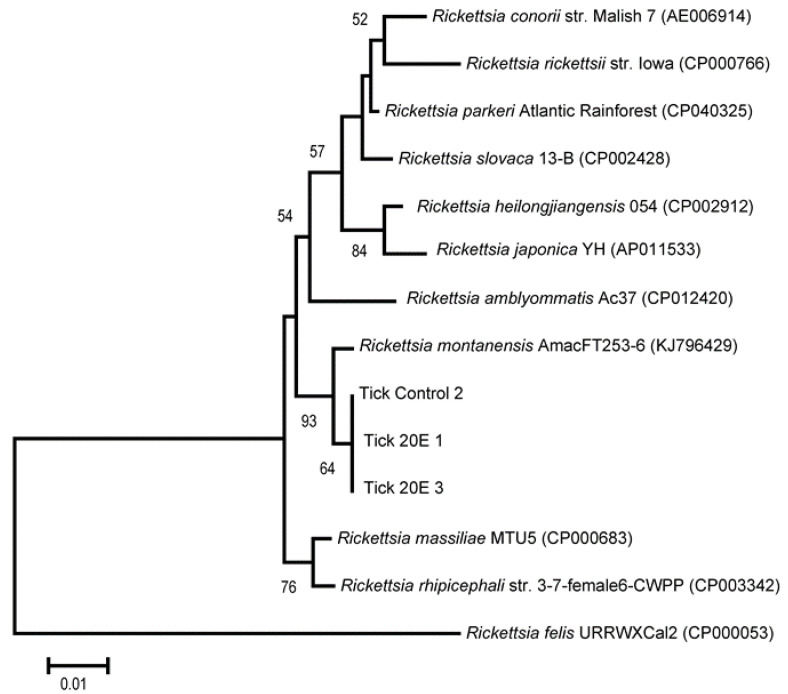
Neighbor-joining tree based on 23-5S gene sequences showing the relationship between sequences from *Dermacentor variabilis* and sequences of other closely related *Rickettsia* sp. Sequences labelled as Tick 20E 1, Tick 20E 3, and Tick Control 2 originated from our *Dermacentor variabilis* samples. The sequences were aligned using the Clustal W algorithm. The numbers at branch nodes show bootstrap percentages (≥50%) based on 1000 resampling. The scale bar on the tree represents 0.01 substitutions per nucleotide position.

A phylogenetic tree was constructed based on the *Rickettsia* 23S-5S intergenic spacer (IGS) region sequences, including comparisons to closely related species of *Rickettsia* spp. (Figure 5). All three of the *Rickettsia* sequences from the ticks used in this study were 100% identical and were clustered into a phylogeny closely related to *Rickettsia montanensis*. All three sequences had 99.4% identity to the *Rickettsia montanensis* AmacFT253-6 (KJ796429) endosymbiont of *D. variabilis*.

## 4. Discussion

Female reproduction in *D. variabilis* begins when unfed, adult males and females acquire the same animal host at the same time. Both attach and blood feed, the females to the part-fed condition and the males to completion. Once the female reaches the part-fed condition, she releases a male sex attractant, and the blood fed male detaches, finds the female and inserts its mouthparts into the female genital pore. This stimulates the male to produce and transfer a spermatophore to the female genital track [10,16,17,29]; reviewed by Roe et al. [18]. The spermatophore transfer includes a gonadotropin that initiates female feeding to repletion (the “big sip”) and egg development. After the “big sip”, the female detaches from the host, falls to the ground, eventually develops and deposits her full complement of eggs, and dies.

**Figure 6:**
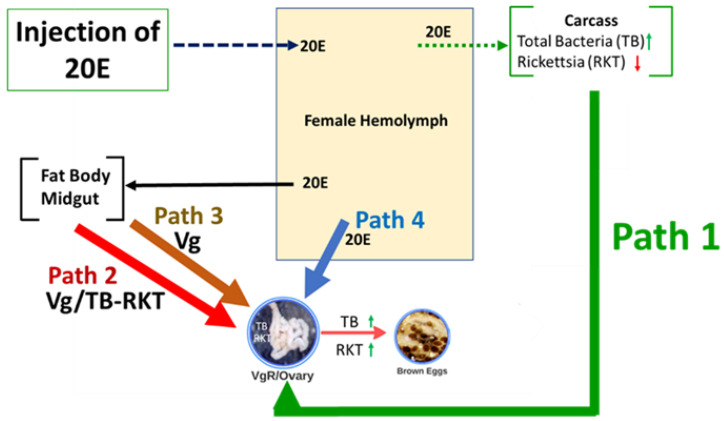
Possible mechanisms for ecdysteroid signaling affecting the microbiome in part-fed, virgin adult *Dermacentor variabilis*. The up and down arrows are indicating changes in bacteria density associated with 20E injection. 20E, 20-hydroxyecdysone; Path, pathway; Vg, vitellogenin; VgR, Vg receptor; Vn, vitellin.

The male gonadotropin transferred to part-fed *D. variabilis* females also stimulates the synganglion to produce and release into the hemolymph a peptidic ecdysteroidaltropic hormone (EDTH). EDTH initiates the epidermal synthesis and release of 20E into the hemolymph, and 20E initiates yolk (Vg) deposition in the eggs. When we injected 20E directly into the hemolymph of part-fed, virgin female adults (attached to a rabbit), this stimulated Vg synthesis and its release into the hemolymph and uptake into eggs (Figure 6) similar to mating. The oocytes become brown (shown in Figure 6) because Vg and Vn are heme-glycolipoproteins; heme is naturally brown in color. Vn containing heme provides the nutrients needed for embryo development [16,19,30]. Ticks cannot synthesize heme and acquire the heme from the host blood. Thompson et al. [29] and Mitchell et al. [17] found that the 20E injection initiated vitellogenesis, the appearance of VgR, and the production of brown eggs and normal ovary weights. However, this treatment did not initiate blood feeding to repletion, drop-off from the host or oviposition; these developmental processes require the transfer of a spermatophore.

There is some evidence in the scientific literature that hormones can affect bacteria growth in animals. For example, estradiol and progesterone stimulated the growth of *Lactobacillus* spp., *Streptococcus* spp., and *Escherichia* in aspirated follicular fluid collected during in vitro fertilization in humans [45]. In insects, *Wolbachia* were important for stimulating juvenile hormone- and 20E-responsive fat body and ovarian follicular cells [46]. The influence of tick hormones on the tick microbiome has not been studied.

There is evidence in insects that bacteria and viruses can move from the female into her eggs. Maternal transmission of *Wolbachia* bacteria was suggested when they were found in insect germline precursors known as “pole cells” [47,48,49]. Herren et al. [50] found the maternal transmission of *Spiroplasma* in the fruit fly, *Drosophila melanogaster*, when the bacteria were endocytosed along with Vg from the intercellular space surrounding ovarian follicle cells into oocytes. In honey bees, *Apis mellifera*, there was evidence suggesting Vg was used for the transport of *E. coli* cell-wall fragments into eggs [51]. In the small brown planthopper insect, *Laodelphax striatellus*, *Wolbachia* passed through the nutritive cord with the help of the host Vg transportation system; the bacteria then entered into the tropharium at the anterior of the ovary [52]. There is also evidence that insect-associated viruses can “hitch-a-ride” on Vg to enter eggs [53]. The vertical transmission of the rice stripe virus (RSV) in the small brown planthopper occurred when RSV hitchhiked by binding to Vg, entering the oocytes through VgR-mediated endocytosis [11]. Specific RSV surface peptides might have facilitated this process [54]. Tufail and Takeda [55,56] suggested that the maternal transmission of endoparasitic microbes in insects was associated with yolk production.

In ticks, transovarial transmission of tick-borne pathogens is an important mechanism for their maintenance in natural populations [9]. Rickettsiae bacteria in ticks are passed from one generation to the next from the female to her eggs [5]. This vertical transmission is critical for maintaining infected tick populations in *D. variabilis* and the horizontal transmission of pathogenic SFG *Rickettsia* [10]. Although the exact mechanism for this transfer to eggs has not been studied, it is known in general that rickettsiae pass from one host cell to an adjacent cell by actin-mediated bridging [25]. There has been some work with the movement of protozoa into tick eggs. Silencing VgR in the tick, *H. longicornis*, blocked the transmission of the protozoan, *Babesia gibsoni*, from the midgut into oocytes [22] and at the same time prevented Vn deposition. Hussein et al. [28] also found that silencing VgR in *R. microplus* blocked the transmission of *B. bovis* and egg development. Based on these tick studies and those in insects, it is reasonable to hypothesize that bacterial pathogens might move from the mother tick to her eggs using the Vg−VgR transfer system. Since the Vg−VgR pathway is under endocrine control, tick hormones might be involved in this movement either directly or indirectly.

Using 16S rRNA gene amplification of the V3–V4 hypervariable region of the bacteria genome and Illumina sequencing, we examined the bacteria composition of part-fed (virgin) *D. variabilis* adults attached to a rabbit host before and after injection with 20E (compared to buffer injected controls). The injections were made into part-fed (virgin) female ticks attached to the rabbit host since this treatment stimulated egg development (Figure 6) but not blood feeding, and the exclusive effect of the hormone treatment on the microbiome could be determined. In duplicate samples, we found *Rickettsia montanensis* in both the ovaries and carcass (Figure 1 and Figure 5; Table 1). The presence of this non-pathogenic *R. montanensis* was considered a reasonable proxy for the study of a possible role of tick hormones on pathogenic *Rickettsia* spp. proliferation and transovarial movement.

To examine further the tick’s hormonal influence on its microbiome, we used qPCR to quantify *R. montanensis* density in the carcass versus ovary before and after injection of 20E. The 20E decreased *R. montanensis* density 3.5-fold in the carcass and increased its density in the ovaries 6.8-fold (Figure 3 and Figure 6). Furthermore, when we compared *R. montanensis* density for the ovary versus carcass for the buffer injected ticks, the density was higher in the carcass (2.4-fold) but after 20E injection, the reverse occurred, *R. montanensis* was 4.7-fold higher in the ovary than carcass (Figure 4). Clearly, the bacteria levels are increasing in the ovary and decreasing, in comparison, in the carcass. One possible mechanism for this increase is the use of the Vg/VgR pathway for *R. montanensis* migration to the ovary (Figure 6, Pathway 2). The 20E injection initiates this pathway and egg development but not blood feeding, and there is evidence in insects that bacteria and viruses and in ticks, protozoa move into eggs only when the Vg transfer pathway is active (reviewed earlier). On the other hand, traditional thinking is that *Rickettsia* spp. move only by cell-to-cell contact, not outside of cells. Other possible explanations for proliferation in the ovary in our studies are the following (illustrated in Figure 6): (i) *Rickettsia* migrated from the carcass to the ovaries independent of the Vg/VgR transport system by cell-to-cell interactions (Pathway 1); (ii) Vg itself when deposited as Vn in the eggs provided a nutritive source for *R. montanensis* replication and/or was a signal for bacteria proliferation in the ovary (Pathway 3); (iii) 20E in the hemolymph directly signaled bacteria replication in the ovary (Pathway 4); or (iv) any combinations of paths 1–4. Surprisingly, there have been no studies to determine if *Rickettsia* spp. are found free-living in hemolymph plasma, which would be necessary if the Vg/VgR pathway is being used for their transport into the ovary. Vg is extracellular in the hemolymph plasma and endocytosed directly from the plasma into eggs. The presence of *R. montanensis* in hemocytes also has not been investigated.

The 20E injections increased the overall bacteria density in both the carcass and ovaries. The microbiota analysis found Pseudomonadaceae in the carcass after the 20E injection which were not found in the control or in the ovaries. There also was a greater proportionality of the genus *Pseudomonas* and other bacteria in the carcass, compared to the control; this appearance might explain at least in part the increased total bacteria density in the carcass. More research will be needed to understand the functional significance of this 20E-induced change in the carcass *Pseudomonas*. This genus was found in blacklegged ticks in the eastern USA [57,58]. In *I. scapularis*, *Rickettsia* and other bacteria were lower in number in *Anaplasma phagocytophilum*-infected ticks, whereas *Pseudomonas* increased in *A. phagocytophilum*-infected ticks, compared to the uninfected [59]. The role of *Pseudomonas* in the microbiome of ticks remains to be explained.

Illumina sequencing found that *Francisella* bacteria, along with *R. montanensis,* were the predominant bacteria in carcass and ovaries both before and after the 20E treatment. Recent surveys showed that *D. variabilis* was infected with this intracellular *Francisella* [60]. *Francisella* is vertically-inherited in *D. variabilis* through transovarial transmission [61]. The development of vertical transmission requires a bottleneck effect where barely a few bacteria are moved into developing eggs [62]. *Francisella* in ticks are a source of B-vitamins [61,63] which might promote tick fitness.

## 5. Conclusions

In summary, we provide the first evidence that the tick microbiome is responsive to the tick hormone, 20-hydroxyecdysone, that also regulates vitellogenesis and the development of fully developed eggs in part-fed (virgin) female *D. variabilis*. Several bacteria species were responsive to 20E which was different between the carcass and ovaries. There was a reduction in density for *R. montanensis* in the carcass and an increase in the ovaries associated with the 20E treatment and in the absence of blood feeding. *Francisella* and *R. montanensis* were the predominant bacteria based on proportionality in the carcass and ovary in the 20E treatments and controls. The mechanism of how *Rickettsia* sp. are responsive to a tick hormone needs further investigation. Multiple possible mechanisms for the proliferation of *R. montanensis* in the ovaries were proposed in our model.

## Figures and Tables

**Table 1:** Summary of sequencing read data.

20E Ovaries		Control Ovaries		20E Carcass		Control Carcass	Taxonomy	
Reads	%	Reads	%	Reads	%	Reads	%	
22,507.5	43.82	12,803.5	41.24	10,135	32.51	38,957	64.38	*Rickettsia*
26,622.5	51.53	15,504.5	49.25	8686	27.95	17,403.5	29.30	*Francisella*
208.5	0.41	1.5	0.004	7425.5	23.76	5	0.008	*Pseudomonas*
665	1.28	1355	4.41	661.5	2.08	939.5	1.58	Unassigned #
1525	2.95	1577.5	5.09	4246.5	13.71	2861	4.74	Others *

# Unassigned bacteria. * “Other taxa” refers to all the taxa with relative abundance < 1% over the total number of reads.

## References

[1] CDC (2018). Tickborne Diseases of the United States: A Reference Manual for Health Care Providers.

[2] Werner S.L., Banda B.K., Burnsides C.L., Stuber A.J. (2019). Zoonosis: Update on existing and emerging vector-borne illnesses in the USA. Curr. Emerg. Hosp. Med. Rep..

[3] Macaluso K., Paddock C., Sonenshine D.E., Roe R.M. (2014). Tick-borne spotted fever group ricketsioses and *Rickettsia* species. Biology of Ticks.

[4] Madison-Antenucci S., Kramer L.D., Gebhardt L.L., Kauffman E. (2020). Emerging tick-borne diseases. Clin. Microbiol. Rev..

[5] Parola P., Paddock C.D., Raoult D. (2005). Tick-borne Rickettsioses around the world: Emerging diseases challenging old concepts. Clin. Microbiol. Rev..

[6] Parola P., Paddock C.D., Socolovschi C., Labruna M.B., Mediannikov O., Kernif T., Abdad M.Y., Stenos J., Bitam I., Fournier P.-E. (2013). Update on tick-borne rickettsioses around the world: A geographic approach. Clin. Microbiol. Rev..

[7] Piotrowski M., Rymaszewska A. (2020). Expansion of tick-borne rickettsioses in the world. Microorganisms.

[8] Wood H., Artsob H. (2012). Spotted fever group Rickettsiae: A brief review and a Canadian perspective. Zoonoses Public Health.

[9] Randolph S.E. (1994). The relative contributions of transovarial and transstadial transmission to the maintenance of tick-borne diseases. Lyme Borreliosis.

[10] Sonenshine D.E., Hynes W.L., Ceraul S.M., Mitchell R., Benzine T. (2005). Host blood proteins and peptides in the midgut of the tick *Dermacentor variabilis* contributeto bacterial control. Exp. Appl. Acarol..

[11] He K., Lin K., Ding S., Wang G., Li F. (2019). The vitellogenin receptor has an essential role in vertical transmission of rice stripe virus during oogenesis in the small brown plant hopper. Pest Manag. Sci..

[12] Germond J., Brownluedi M., Walker P., Debony E., Wahli W. (1984). Sequence homologies within the 5’end region of the 4 estrogen-controlled vitellogenin genes in *Xenopus* and chicken. Experientia.

[13] Spieth J., Nettleton M., Zucker-Aprison E., Lea K., Blumenthal T. (1991). Vitellogenin motifs conserved in nematodes and vertebrates. J. Mol. Evol..

[14] Wahli W. (1988). Evolution and expression of vitellogenin genes. Trends Genet..

[15] Hamblin M.T., Marx J.L., Wolfner M.F., Hagedorn H.H. (1987). The vitellogenin gene family of *Aedes aegypti*. Mem. Inst. Oswaldo Cruz.

[16] Khalil S.M., Donohue K.V., Thompson D.M., Jeffers L.A., Ananthapadmanaban U., Sonenshine D.E., Mitchell R.D., Roe R.M. (2011). Full-length sequence, regulation and developmental studies of a second vitellogenin gene from the American dog tick, *Dermacentor variabilis*. J. Insect Physiol..

[17] Mitchell R.D., Ross E., Osgood C., Sonenshine D.E., Donohue K.V., Khalil S.M., Thompson D.M., Roe R.M. (2007). Molecular characterization, tissue-specific expression and RNAi knockdown of the first vitellogenin receptor from a tick. Insect Biochem. Mol. Biol..

[18] Roe R.M., Donohue K.V., Khalil S.M., Bissinger B.W., Zhu J., Sonenshine D.E., Sonenshine D.E., Michael R.R. (2014). Hormonal Regulation of Metamorphosis and Reproduction in Ticks.

[19] Xavier M.A., Tirloni L., Pinto A.F., Diedrich J.K., Yates J.R., Mulenga A., Logullo C., da Silva Vaz I., Seixas A., Termignoni C. (2018). A proteomic insight into vitellogenesis during tick ovary maturation. Sci. Rep..

[20] Gulia-Nuss M., Nuss A.B., Meyer J.M., Sonenshine D.E., Roe R.M., Waterhouse R.M., Sattelle D.B., De La Fuente J., Ribeiro J.M., Megy K. (2016). Genomic insights into the *Ixodes scapularis* tick vector of Lyme disease. Nat. Commun..

[21] Roe R.M., Donohue K.V., Khalil S.M., Sonenshine D.E. (2008). Hormonal regulation of metamorphosis and reproduction in ticks. Front. Biosci..

[22] Boldbaatar D., Battsetseg B., Matsuo T., Hatta T., Umemiya-Shirafuji R., Xuan X., Fujisaki K. (2008). Tick vitellogenin receptor reveals critical role in oocyte development and transovarial transmission of *Babesia* parasite. Biochem. Cell Biol..

[23] Smith A.D., Kaufman W.R. (2013). Molecular characterization of the vitellogenin receptor from the tick, *Amblyomma hebraeum* (Acari: Ixodidae). Insect Biochem. Mol. Biol..

[24] Seixas A., Alzugaray M.F., Tirloni L., Parizi L.F., Pinto A.F.M., Githaka N.W.o., Konnai S., Ohashi K., Yates J.R., Termignoni C. (2018). Expression profile of *Rhipicephalus microplus* vitellogenin receptor during oogenesis. Ticks Tick Borne Dis..

[25] Gouin E., Gantelet H., Egile C., Lasa I., Ohayon H., Villiers V., Gounon P., Sansonetti P., Cossart P. (1999). A comparative study of the actin-based motilities of the pathogenic bacteria *Listeria monocytogenes*, *Shigella flexneri* and *Rickettsia conorii*. J. Cell Sci..

[26] Sonenshine D.E., Macaluso K.R. (2017). Microbial invasion vs. tick immune regulation. Front. Cell. Infect. Microbiol..

[27] Solano-Gallego L., Sainz Á., Roura X., Estrada-Peña A., Miró G. (2016). A review of canine babesiosis: The European perspective. Parasites Vectors.

[28] Hussein H.E., Johnson W.C., Taus N.S., Suarez C.E., Scoles G.A., Ueti M.W. (2019). Silencing expression of the *Rhipicephalus microplus* vitellogenin receptor gene blocks *Babesia bovis* transmission and interferes with oocyte maturation. Parasit Vectors.

[29] Thompson D.M., Khalil S.M., Jeffers L.A., Ananthapadmanaban U., Sonenshine D.E., Mitchell R.D., Osgood C.J., Apperson C.S., Roe R.M. (2005). In vivo role of 20-hydroxyecdysone in the regulation of the vitellogenin mRNA and egg development in the American dog tick, *Dermacentor variabilis* (Say). J. Insect Physiol..

[30] Rosell R., Coons L.B. (1992). The role of the fat body, midgut and ovary in vitellogenin production and vitellogenesis in the female tick, *Dermacentor variabilis*. Int. J. Parasitol..

[31] Mitchell R.D., Sonenshine D.E., Pérez de León A.A. (2019). Vitellogenin receptor as a target for tick control: A mini-review. Front. Physiol..

[32] Sonenshine D.E. (1991). Biology of Ticks.

[33] Ponnusamy L., Gonzalez A., Van Treuren W., Weiss S., Parobek C.M., Juliano J.J., Knight R., Roe R.M., Apperson C.S., Meshnick S.R. (2014). Diversity of Rickettsiales in the microbiome of the lone star tick, *Amblyomma americanum*. Appl. Environ. Microbiol..

[34] Caporaso J.G., Kuczynski J., Stombaugh J., Bittinger K., Bushman F.D., Costello E.K., Fierer N., Pena A.G., Goodrich J.K., Gordon J.I. (2010). QIIME allows analysis of high-throughput community sequencing data. Nat. Methods.

[35] Edgar R.C., Haas B.J., Clemente J.C., Quince C., Knight R. (2011). UCHIME improves sensitivity and speed of chimera detection. Bioinformatics.

[36] DeSantis T.Z., Hugenholtz P., Larsen N., Rojas M., Brodie E.L., Keller K., Huber T., Dalevi D., Hu P., Andersen G.L. (2006). Greengenes, a chimera-checked 16S rRNA gene database and workbench compatible with ARB. Appl. Environ. Microbiol..

[37] Caporaso J.G., Bittinger K., Bushman F.D., DeSantis T.Z., Andersen G.L., Knight R. (2010). PyNAST: A flexible tool for aligning sequences to a template alignment. Bioinformatics.

[38] Lazarevic V., Gaïa N., Girard M., Schrenzel J. (2016). Decontamination of 16S rRNA gene amplicon sequence datasets based on bacterial load assessment by qPCR. BMC Microbiol..

[39] Browning R., Adamson S., Karim S. (2014). Choice of a stable set of reference genes for qRT-PCR analysis in *Amblyomma maculatum* (Acari: Ixodidae). J. Med. Entomol..

[40] Kakumanu M.L., Ponnusamy L., Sutton H.T., Meshnick S.R., Nicholson W.L., Apperson C.S. (2016). Development and validation of an improved PCR method using the 23S-5S intergenic spacer for detection of rickettsiae in *Dermacentor variabilis* ticks and tissue samples from humans and laboratory animals. J. Clin. Microbiol..

[41] Larkin M.A., Blackshields G., Brown N.P., Chenna R., McGettigan P.A., McWilliam H., Valentin F., Wallace I.M., Wilm A., Lopez R. (2007). Clustal W and Clustal X version 2.0. Bioinformatics.

[42] Saitou N., Nei M. (1987). The neighbor-joining method: A new method for reconstructing phylogenetic trees. Mol. Biol. Evol..

[43] Kimura M. (1980). A simple method for estimating evolutionary rates of base substitutions through comparative studies of nucleotide sequences. J. Mol. Evol..

[44] Kumar S., Stecher G., Li M., Knyaz C., Tamura K. (2018). MEGA X: Molecular evolutionary genetics analysis across computing platforms. Mol. Biol. Evol..

[45] Pelzer E.S., Allan J.A., Theodoropoulos C., Ross T., Beagley K.W., Knox C.L. (2012). Hormone-dependent bacterial growth, persistence and biofilm formation–a pilot study investigating human follicular fluid collected during IVF cycles. PLoS ONE.

[46] Negri I. (2012). *Wolbachia* as an “infectious” extrinsic factor manipulating host signaling pathways. Front. Endocrinol..

[47] Bressac C., Rousset F. (1993). The reproductive incompatibility system in *Drosophila simulans*: DAPI-staining analysis of the *Wolbachia* symbionts in sperm cysts. J. Invertebr. Pathol..

[48] Hadfield S.J., Axton J.M. (1999). Reproduction: Germ cells colonized by endosymbiotic bacteria. Nature.

[49] Clark M.E., Veneti Z., Bourtzis K., Karr T.L. (2003). *Wolbachia* distribution and cytoplasmic incompatibility during sperm development: The cyst as the basic cellular unit of CI expression. Mech. Dev..

[50] Herren J.K., Paredes J.C., Schüpfer F., Lemaitre B. (2013). Vertical transmission of a *Drosophila* endosymbiont via cooption of the yolk transport and internalization machinery. MBio.

[51] Salmela H., Amdam G.V., Freitak D. (2015). Transfer of immunity from mother to offspring is mediated via egg-yolk protein vitellogenin. PLoS Pathog..

[52] Guo Y., Hoffmann A., Xu X.-Q., Mo P.-W., Huang H.-J., Gong J.-T., Ju J.-F., Hong X.-Y. (2018). Vertical transmission of *Wolbachia* is associated with host vitellogenin in *Laodelphax striatellus*. Front. Microbiol..

[53] Huo Y., Liu W., Zhang F., Chen X., Li L., Liu Q., Zhou Y., Wei T., Fang R., Wang X. (2014). Transovarial transmission of a plant virus is mediated by vitellogenin of its insect vector. PLoS Pathog..

[54] Huo Y., Yu Y., Liu Q., Liu D., Zhang M., Liang J., Chen X., Zhang L., Fang R. (2019). Rice stripe virus hitchhikes the vector insect vitellogenin ligand-receptor pathway for ovary entry. Philos. Trans. R. Soc. Lond. B Biol. Sci. B.

[55] Tufail M., Takeda M. (2008). Molecular characteristics of insect vitellogenins. J. Insect Physiol..

[56] Tufail M., Takeda M. (2009). Insect vitellogenin/lipophorin receptors: Molecular structures, role in oogenesis, and regulatory mechanisms. J. Insect Physiol..

[57] Van Treuren W., Ponnusamy L., Brinkerhoff R.J., Gonzalez A., Parobek C.M., Juliano J.J., Andreadis T.G., Falco R.C., Ziegler L.B., Hathaway N. (2015). Variation in the microbiota of Ixodes ticks with regard to geography, species, and sex. Appl. Environ. Microbiol..

[58] Thapa S., Zhang Y., Allen M.S. (2019). Bacterial microbiomes of Ixodes scapularis ticks collected from Massachusetts and Texas, USA. BMC Microbiol..

[59] Abraham N.M., Liu L., Jutras B.L., Yadav A.K., Narasimhan S., Gopalakrishnan V., Ansari J.M., Jefferson K.K., Cava F., Jacobs-Wagner C. (2017). Pathogen-mediated manipulation of arthropod microbiota to promote infection. Proc. Natl. Acad. Sci. USA.

[60] Travanty N.V., Ponnusamy L., Kakumanu M.L., Nicholson W.L., Apperson C.S. (2019). Diversity and structure of the bacterial microbiome of the American dog tick, *Dermacentor variabilis,* is dominated by the endosymbiont *Francisella*. Symbiosis.

[61] Duron O., Morel O., Noël V., Buysse M., Binetruy F., Lancelot R., Loire E., Ménard C., Bouchez O., Vavre F. (2018). Tick-bacteria mutualism depends on B vitamin synthesis pathways. Curr. Biol..

[62] Mira A., Moran N.A. (2002). Estimating population size and transmission bottlenecks in maternally transmitted endosymbiotic bacteria. Microb. Ecol..

[63] Gerhart J.G., Moses A.S., Raghavan R. (2016). A Francisella-like endosymbiont in the Gulf Coast tick evolved from a mammalian pathogen. Sci. Rep..

